# Oxidative Stress and Hemostatic Parameters in Patients With Nephrolithiasis Before and After Ureteroscopic Lithotripsy

**DOI:** 10.3389/fphys.2019.00799

**Published:** 2019-06-21

**Authors:** Paweł Woźniak, Bogdan Kontek, Bartosz Skalski, Anna Król, Waldemar Różański, Beata Olas

**Affiliations:** ^1^2nd Department of Urology, Medical University of Łódź, Łódź, Poland; ^2^Department of General Biochemistry, Faculty of Biology and Environmental Protection, University of Łódź, Łódź, Poland

**Keywords:** hemostasis, nephrolithiasis, oxidative stress, ureteroscopic lithotripsy, extracorporeal shock wave lithotripsy

## Abstract

**Purpose:**

In patients with nephrolithiasis, oxidative stress, especially lipid peroxidation is observed. Moreover, various invasive methods [including extracorporeal shock wave lithotripsy (ESWL)] for treatment of nephrolithiasis may induce not only the oxidative stress, but they may modulate hemostasis. The study was aimed to evaluate the oxidative damages of lipids and proteins in patients with nephrolithiasis (before and after ureteroscopic lithotripsy – URSL). The aim of the present study was also determine selected parameters of hemostasis in these patients.

**Methods:**

56 patients with nephrolithiasis and 49 healthy participants were included: 30 men and 26 women (for patient group); 27 men and 22 women (for healthy group). We measured the level of selected typical two biomarkers of oxidative modification of lipids [such as the production of thiobarbituric acid reactive substances (TBARS) and isoprostane concentration (8-isoPGF_2α_)] and two biomarkers of oxidative damages of proteins (carbonylation and the level of thiol groups) in patients with nephrolithiasis (before and after URSL). The following parameters of hemostasis were measured: blood platelet count, the level of fibrinogen and D-dimer, and coagulation times (the activated partial thromboplastin time (APTT), prothrombin time (PT), and thrombin time (TT) of plasma).

**Results:**

Different levels of plasma lipid peroxidation were observed in patients with nephrolithiasis before URSL and after URSL. However, no such difference in the level of oxidative damage to plasma proteins was observed. In addition, the tested hemostasis parameters were not influenced by the presence of nephrolithiasis, nor by treatment with URSL.

**Conclusion:**

We suggest URSL does not induce the oxidative modifications of plasma proteins and does not change hemostatic parameters in patients with nephrolithiasis.

## Introduction

Oxidative stress causes damage to different biomolecules – lipids, proteins and DNA. Moreover, oxidative stress may induce changes elements of hemostasis (including coagulation process and blood platelet activation) in various diseases. In recent years, various studies demonstrated that oxidative stress exists in patients with nephrolithiasis, however, the mechanism(s) of this process is not well known. It has been previously shown that especially lipid peroxidation [measured by different biomarkers: 8-isoprostaglandin F_2_ (8-isoPGF_2α_) and thiobarbituric acid reactive substances (TBARS)] exists in these patients. Increase of lipid peroxidation was demonstrated in different biological materials (serum, plasma, erythrocytes, and urine) obtained from patients with nephrolithiasis (Khan, 2005, 2012, 2013a,b, 2014; Boonla et al., 2007; Ma et al., 2014; Ceban et al., 2016). In addition, various invasive methods [including extracorporeal shock wave lithotripsy (ESWL)] for treatment of nephrolithiasis may also induce the oxidative stress (Gecit et al., 2012; Garca et al., 2014; Wozniak et al., 2017, 2018) and modulate hemostasis (Wozniak et al., 2017). Modulation of hemostasis was observed 30–240 min after shock wave lithotripsy (SWL) (Hughes et al., 2015) and the day after ESWL (Wozniak et al., 2017).

Ureteroscopic lithotripsy (URSL) is one of the most common operations in urology. Moreover, it is and important that is an effective and safe method for managing ureteral stones (Seitz et al., 2007; Tao et al., 2015). Complications occur in less than 0.1% of cases (Nuttall et al., 2010). However, the effect of URSL on parameters of oxidative stress and hemostasis in patients with nephrolithiasis has not been studied yet. Thus, the main objective of our experiments was to examine the level of selected typical two biomarkers of oxidative modification of lipids (such as the production of TBARS and isoprostane concentration) and two biomarkers of oxidative damages of proteins (carbonylation and the level of thiol groups) in patients with nephrolithiasis (before and after URSL). Moreover, the aim of the present study was also determine selected parameters of hemostasis (blood platelet count, the level of fibrinogen and D-dimer, and coagulation times (the activated partial thromboplastin time (APTT), prothrombin time (PT), and thrombin time (TT) of plasma) in these patients.

## Materials and Methods

### Patients and Samples

The blood samples were collected from 56 patients (30 men and 26 women) whom had been referred to the 2nd Department of Urology, Medical University of Łódź, Poland, for URSL. The exclusion criteria were as follows: the presence of urinary tract infection, other inflammatory or malignant disease, diabetes mellitus, history of heart disease, or chronic kidney disease. Before URSL anatomy of the kidney and excretory path for all patients were investigated by intravenous urography, and no anomalies were found.

A group of 49 healthy individuals (27 men and 22 women) were collected from the hospital from routine controls of health and used as control. They were non-related men and women, that have never been diagnosed with nephrolithiasis or chronic disease and were randomly selected and frequency matched to the cases on age.

The blood and urine samples were collected from the patients before URSL, and 1 day after URSL. The blood samples and the urine samples were taken from patients and healthy participants keeping a balanced diet (meat and vegetables), with similar socio-economic background, using no antioxidant supplementation and any medications (f.e. anti-platelet drugs or anti-inflammatory agents).

Erythrocytes were separated from plasma by centrifugation (2800 × *g* for 10 min). Concentration of hemoglobin was determined by the cyanohemoglobin method using Drabkin’s reagent. Participants provided also first morning void urine samples (50–100 ml), which was kept on ice and processed within 4 h. Plasma samples obtained from the participants were stored at −80°C within 2 h of removal.

The protocol was passed by the Committee for Research on Human Subjects of the Medical University of Łódź RNN/101/13/KE. The protocol was passed by the Committee for Research on Human Subjects of the Medical University of Łódź RNN/101/13/KE. The first, participants provided verbal consent to the researchers, and later participants provided written the documents. Authors had access to identifying participant information.

### Parameters of Oxidative Stress

#### Measurement of Lipid Peroxidation – The Level of 8-isoPGF_2α_

The level of 8-isoPGF_2α_ was estimated in urine samples from control subjects and from patients using an immunoassay kit (Cayman Chemical) according to the manufacturer’s instructions.

#### Measurement of Lipid Peroxidation – The Level of TBARS

Samples of plasma or erythrocytes were transferred to an equal volume of cold 20% (v/v) trichloroacetic acid in 0.6 M HCl and centrifuged at 1200 × *g* for 15 min. One volume of clear supernatant was mixed with 0.2 volume of 0.12 M thiobarbituric acid in 0.26 M Tris (pH 7.0) immersed in a boiling water bath for 15 min. and then absorbance was measured at 535 nm (the SPECTROstar Nano Microplate Reader – BMG LABTECH Germany) (Wachowicz, 1984; Bartosz, 2008). The TBARS concentration was calculated using the molar extinction coefficient (ε = 156,000 M^−1^cm^−1^).

#### Measurement of Protein Carbonylation

The detection of carbonyl groups in proteins was carried out according to Levine et al. (1990) and Bartosz (2008). The carbonyl group concentration was calculated using a molar extinction coefficient (ε = 22,000 M^−1^cm^−1^), and the level of carbonyl groups was expressed as nmol carbonyl groups/mg of protein. Carbonyl content was determined by taking the SPECTROstar Nano Microplate Reader – BMG LABTECH Germany.

#### Measurement of the Level of Thiol Groups

The thiol group content was measured spectrophotometrically (the SPECTROstar Nano Microplate Reader – BMG LABTECH Germany) by absorbance at 412 nm with Ellman’s reagent: 5,5′-dithio-bis-(2-nitrobenzoic acid). The thiol group concentration was calculated using a molar extinction coefficient (ε = 13,600 M^−1^cm^−1^) (Ando and Steiner, 1973a,b; Bartosz, 2008). The level of thiol groups was expressed as nmol thiol groups/mg of plasma protein.

### Parameters of Hemostasis

#### The Measurement of Prothrombin Time

The PT (seconds) was determined coagulometrically (BCS XP Healthcare Diagnostics Siemens, Germany) in citrated samples.

#### The Measurement of Thrombin Time

The TT (seconds) was determined coagulometrically (BCS XP Healthcare Diagnostics Siemens, Germany) in citrated samples.

#### The Measurement of APTT

The APTT (seconds) was determined coagulometrically (BCS XP Healthcare Diagnostics Siemens, Germany) in citrated samples.

#### The Measurement of Blood Platelet Concentration

Blood platelet count was performed using an automated cell counter (Sysmex XN-2000, Sysmex, Japan) in citrated samples. The platelets were measured in units × 10^9^/l.

#### The Measurement of Fibrinogen

Fibrinogen (g/l) concentration (in citrated samples) was measured using an analyzer (BCS XP Healthcare Diagnostics Siemens, Germany).

#### The Measurement of D-Dimer

D-dimer (ng/ml) concentration was determined by an analyser (BCS XP Healthcare Diagnostics Siemens, Germany) in citrated samples.

### Statistical Analysis

The statistical analysis was done by several tests. All the values in this study were expressed as mean ± SD. In order to eliminate uncertain data, Q-Dixon test was performed. Since levels of biomarkers of oxidative stress in studied material did not show normal distribution (Kolmogorov–Smirnov test) the non-parametrical statistical test (Mann-Whitney *U* test) was applied. Reported *p*-values were two-sided. All analyses were completed using STATISTICA 12.

## Results

**FIGURE 1:**
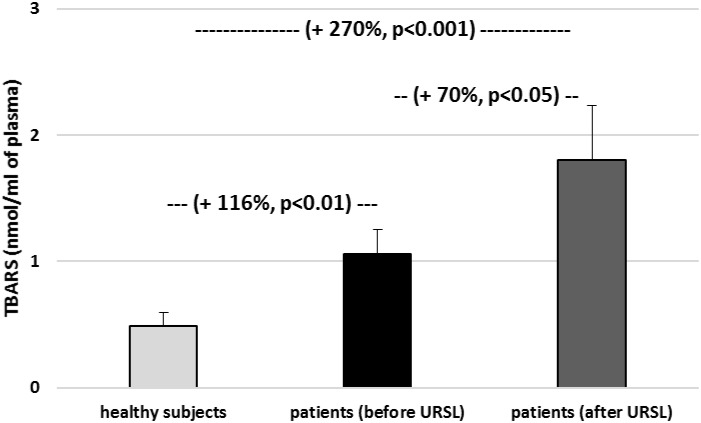
The level of TBARS in plasma in patients with nephrolithiasis (before and after URSL), and in control plasma obtained from healthy volunteers. Results are means ± SD. The statistically analysis was done by Mann–Whitney test (for means ± SD).

**FIGURE 2:**
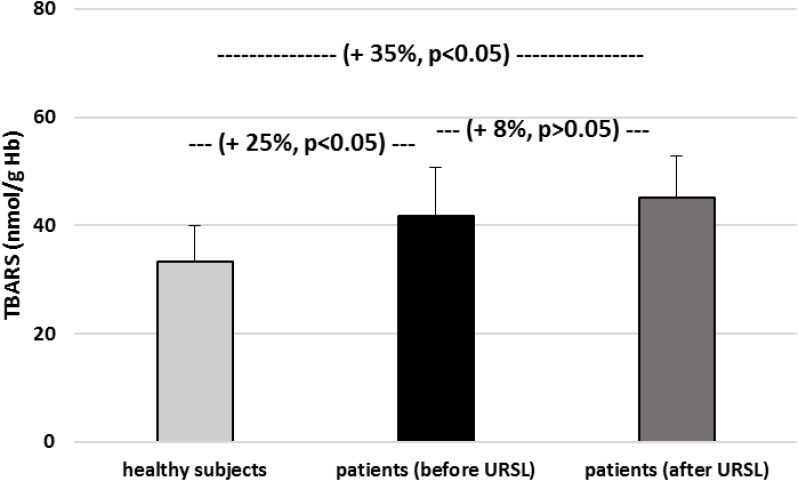
The level of TBARS in erythrocytes in patients with nephrolithiasis (before and after URSL), and in control erythrocytes obtained from healthy volunteers. Results are means ± SD. The statistically analysis was done by Mann–Whitney test (for means ± SD).

As demonstrated on Figure 1, the TBARS level in plasma was significantly increase in patients with nephrolithiasis (before URSL) than in control. In plasma samples from patients with nephrolithiasis (before URSL), the level of TBARS was increase by about 116% compare to control group (Figure 1). The concentration of TBARS in plasma from patients with nephrolithiasis (after URSL) was also higher than in patients with nephrolithiasis (before URSL) and higher than in healthy volunteers (Figure 1). In addition, the concentration of TBARS in erythrocytes in patients with nephrolithiasis (before URSL and after URSL) differed markedly from healthy volunteers (Figure 2). The TBARS content in erythrocytes in patients with nephrolithiasis (before URSL and after URSL) was significantly higher than in controls [and increase – about 25% (before URSL), and about 35% (after URSL)] (Figure 2). The level of TBARS in erythrocytes from patients after URSL was also changed, compared with patients before URSL, however, it was not statistically significant (Figure 2).

**FIGURE 3:**
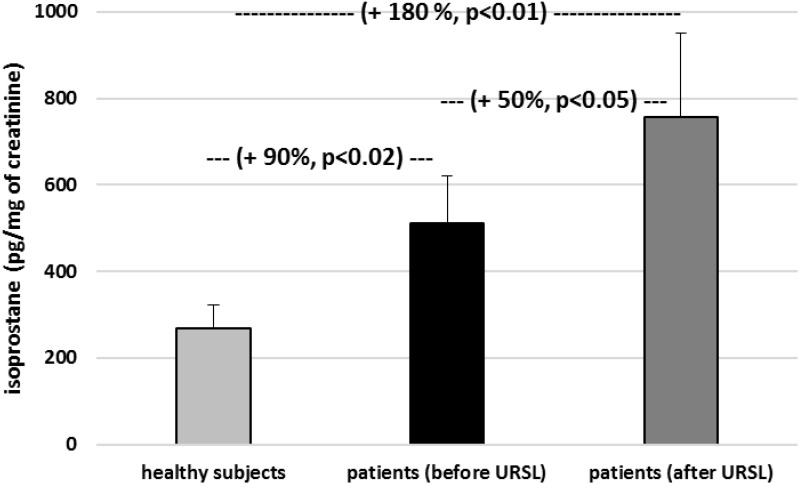
The level of 8-isoPGF_2α_ in urine in patients with nephrolithiasis (before and after URSL), and in control urine obtained from healthy volunteers. Results are means ± SD. The statistically analysis was done by Mann–Whitney test (for means ± SD).

Other experiments showed that the concentration of isoprostane in urine from patients (before and after URSL) was found to be higher than the concentration of isoprostane in urine obtained from healthy volunteers (Figure 3). The concentration of isoprostane in urine from patients (after URSL) was also higher than in patients (before URSL) (Figure 3).

**FIGURE 4:**
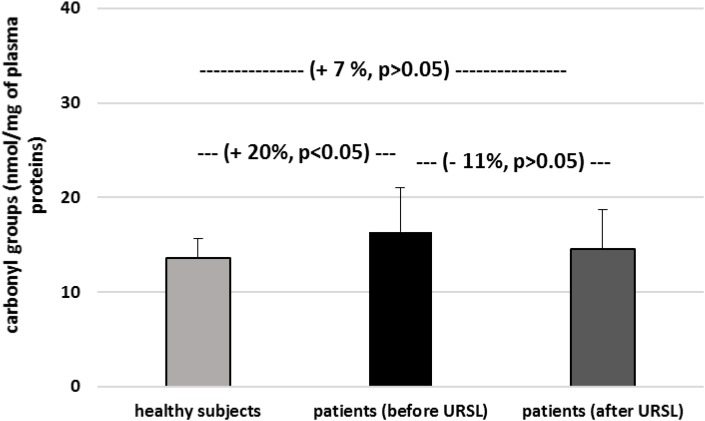
The level of carbonyl groups in plasma proteins in patients with nephrolithiasis (before and after URSL), and in control plasma proteins obtained from healthy volunteers. Results are means ± SD. The statistically analysis was done by Mann–Whitney test (for means ± SD).

**FIGURE 5:**
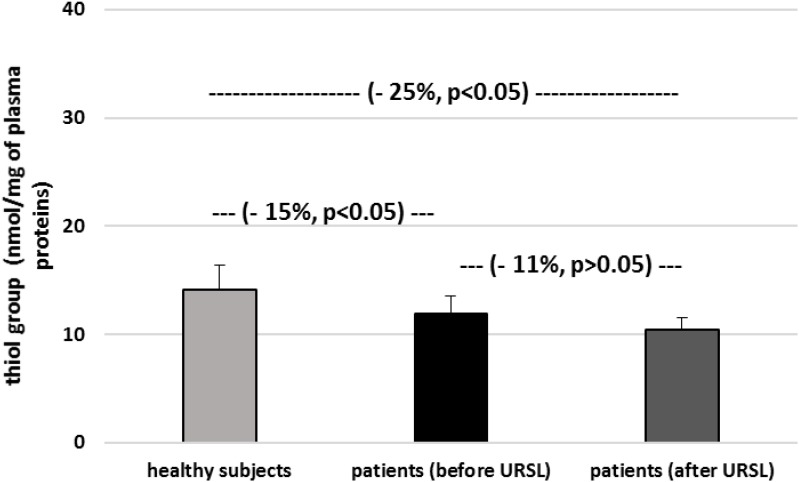
The level of thiol groups in plasma proteins in patients with nephrolithiasis (before and after URSL), and in control plasma proteins obtained from healthy volunteers. Results are means ± SD. The statistically analysis was done by Mann–Whitney test (for means ± SD).

The level of carbonyl groups in plasma proteins from patients with nephrolithiasis (before and after URSL) was found to be higher than the level of carbonyl groups in plasma proteins obtained from healthy volunteers (Figure 4). On the other hand, the level of carbonyl groups in proteins from patients (after URSL) was lower than in proteins from patients (before URSL) (Figure 4). However, this change (in the concentration of carbonyl groups) was not statistically significant (Figure 4). Figure 5 demonstrates that the level of thiol groups in proteins from patients (after URSL) was slightly lower than in proteins from patients (before URSL), but not statistically significant.

**FIGURE 6:**
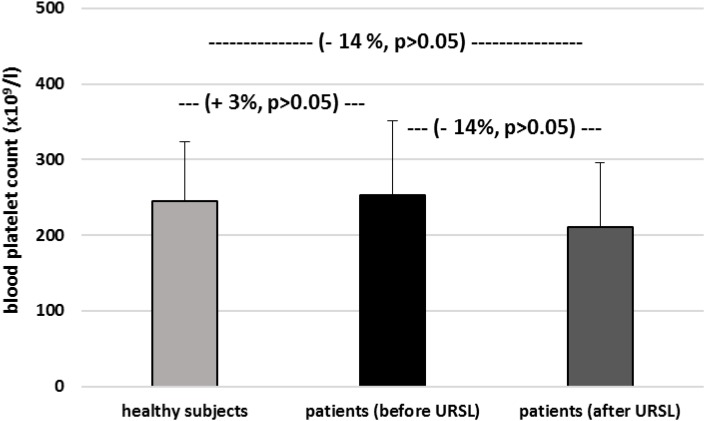
The level of blood platelets in patients with nephrolithiasis (before and after URSL), and in from healthy volunteers. Results are means ± SD. The statistically analysis was done by Mann–Whitney test (for means ± SD).

**FIGURE 7:**
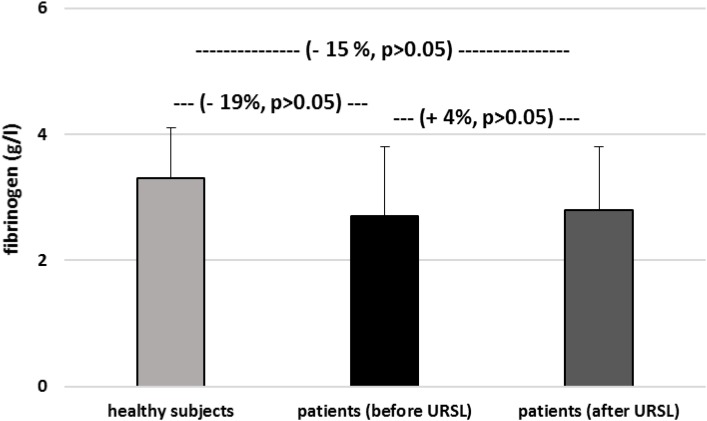
The level of fibrinogen in plasma from patients with nephrolithiasis (before and after URSL), and in control plasma obtained from healthy volunteers. Results are means ± SD. The statistically analysis was done by Mann–Whitney test (for means ± SD).

**FIGURE 8:**
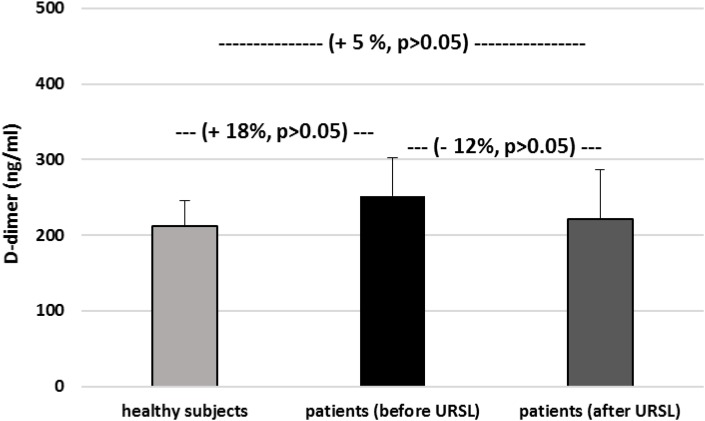
The level of D-dimer in plasma in patients with nephrolithiasis (before and after URSL), and in control plasma obtained from healthy volunteers. Results are means ± SD. The statistically analysis was done by Mann–Whitney test (for means ± SD).

**FIGURE 9:**
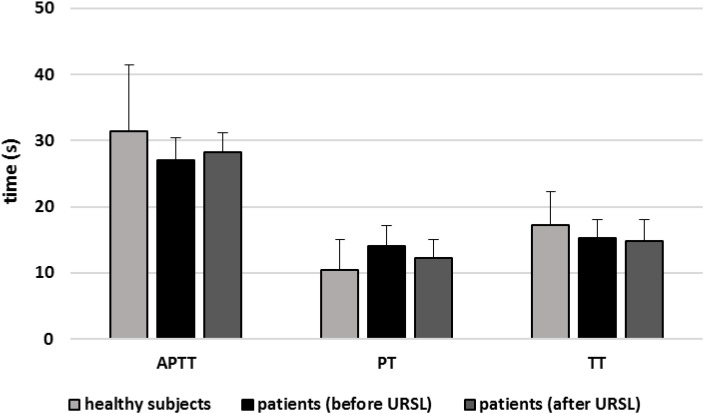
The activated partial thromboplastin time (APTT), prothrombin time (PT), and thrombin time (TT) of plasma in patients with nephrolithiasis (before and after URSL), and in control plasma obtained from healthy volunteers. Results are given as means ± SD. The statistical analysis was performed using the Mann–Whitney test (for all times: *p* > 0.05 – patients [(before/after URSL] *versus* healthy subjects; *p* > 0.05 patients [after ESWL] *versus* patients [(before URSL]).

Figure 6–9 demonstrate parameters of hemostasis in patients with nephrolithiasis before and after URSL. For example, blood platelet counts were (about 14%) lower in patients after URSL than in healthy subjects or in patients before URSL, but these changes were not statistically significant (Figure 6). Neither, fibrinogen, D-dimer and coagulation times (APTT, PT, and TT) were influenced by the presence of nephrolithiasis, nor by treatment with URSL (Figure 7–9).

Moreover, we did not observe the difference in concentrations of biomarkers of oxidative stress between the group of men and the group of women.

## Discussion

Lipids and proteins are major targets for oxidants. Oxidative stress itself is known to play a role in nephrolithiasis through the action of free radicals, which are believed to initiate the inflammation process and induce renal cellular injury (Khan, 2005, 2012, 2013a,b, 2014; Boonla et al., 2007; Ma et al., 2014; Ceban et al., 2016). In addition, increased oxidative stress may also be correlated with kidney stone development (Gaspar et al., 2010). For example, oxidative stress has been reported in the erythrocytes of patients with calcium oxalate stones, resulting in renal tubular damage and increases in stone size (Ma et al., 2014). Other studies suggest that urinary levels of oxalate and citric oxide may be correlated with lipid peroxidation (Huang et al., 2003). The present study by using different biomarkers of oxidative stress – damages of lipids, including the change of TBARS (in plasma and erythrocytes) and the change of isoprostane level in urine provides evidence that in patients with nephrolithiasis, oxidative stress – lipid peroxidation occurs. It should be underlined that in our present experiments tested samples from patients were taken before and after URSL. It is very important, because various drugs, which are used during treatment (i.e., surgery) may induce oxidative stress in different tissues, blood cells and plasma (Wang et al., 2017). However, Ceban et al. (2016) studied oxidant and antioxidant status in the blood of patients with complicated urolithiasis pre- and post-surgery. Oxidant and antioxidant status was measured also by various parameters, including the level of thiol groups and activity of different antioxidant enzymes (i.e., glutathione dismutase and glutathione reductase). Experiments of these authors demonstrated that the surgical treatment of complicated urolithiasis leads a decrease of the oxidative stress and an increase in potential of antioxidant status. On the other hand, *in vitro* and *in vivo* experiments show that increased oxidative stress is associated with kidney stone development (Khan, 2005, 2012, 2013a,b, 2014; Boonla et al., 2007; Ma et al., 2014; Ceban et al., 2016). Our earlier results indicate that ESWL also induces the oxidative stress (measured by the level of carbonyl groups in plasma proteins in patients with nephrolithiasis) and modulates hemostasis in these patients (Wozniak et al., 2017). A key novel finding of this experiment is that patients with nephrolithiasis undergoing other method for treatment – URSL, experience an increase in lipid peroxidation (measured by two typical biomarkers: TBARS and isoprostanes), as compared to healthy volunteers. Moreover, changes in lipid peroxidation were found between patients before URSL and patients who had completed treatment.

Hemostasis is thought to be modulated by oxidative stress (Nowak et al., 2010). However, in the present study, no changes in oxidative damage to plasma proteins, indicated by the levels of thiol groups and carbonyl groups, were observed in the nephrolithiasis patients before URSL and after URSL. In addition, a significant novel finding is that URSL does not appear to induce changes in hemostasis, measured by various typical parameters, including blood platelet count, fibrinogen concentration and coagulation times in these patients. The only possible explanation for this lack of observed change was that no oxidative damage had occurred to the plasma proteins. Hughes et al. (2015) also report that treatment of solitary kidney stones by SWL does not appear to influence the biochemical parameters of hemostasis, measured by coagulation times (APTT and PT).

This present study is the first to examine the effect of URSL on parameters of oxidative stress and hemostasis in patients with nephrolithiasis. Our findings indicate that URSL does not induce any oxidative modification in plasma proteins nor does it change the hemostatic parameters in these patients. However, the differences in levels of oxidative stress biomarkers are small, but they are statistically significant. Moreover, it is not clear whether these statistical changes are clinically significant. Therefore, further experiments based on larger groups of patients are needed to more precisely determine its influence on oxidative stress and hemostasis.

## Data Availability

The raw data supporting the conclusions of this manuscript will be made available by the authors, without undue reservation, to any qualified researcher.

## Ethics Statement

The protocol was passed by the Committee for Research on Human Subjects of the Medical University of Łódź RNN/101/13/KE. The first, participants provided verbal consent to the researchers, and later participants provided written the documents. Authors had access to identifying participant information.

## Author Contributions

All authors have made a substantial, direct, and intellectual contribution to the work and approved it for publication.

## Conflict of Interest Statement

The authors declare that the research was conducted in the absence of any commercial or financial relationships that could be construed as a potential conflict of interest.
